# Regulation of body weight and food intake by AGRP neurons during opioid dependence and abstinence in mice

**DOI:** 10.3389/fncir.2022.977642

**Published:** 2022-08-30

**Authors:** Brenton T. Laing, Aishwarya Jayan, Lydia J. Erbaugh, Anika S. Park, Danielle J. Wilson, Yeka Aponte

**Affiliations:** ^1^Neuronal Circuits and Behavior Section, National Institute on Drug Abuse Intramural Research Program, National Institutes of Health, Baltimore, MD, United States; ^2^The Solomon H. Snyder Department of Neuroscience, Johns Hopkins University School of Medicine, Baltimore, MD, United States

**Keywords:** opioid, morphine, dependence, AGRP neurons, feeding, hypothalamus, metabolism, chemogenetics

## Abstract

Dysregulation of body weight maintenance and opioid dependence are often treated as independent disorders. Here, we assessed the effects of both acute and long-term administration of morphine with and without chemogenetic activation of agouti-related peptide (AGRP)-expressing neurons in the arcuate nucleus (ARC^AGRP^ neurons) to elucidate whether morphine and neuronal activation affect feeding behavior and body weight. First, we characterized interactions of opioids and energy deficit in wild-type mice. We observed that opioid administration attenuated both fasting-induced refeeding and ghrelin-stimulated feeding. Moreover, antagonism of opioid receptors blocked fasting-induced refeeding behavior. Next, we interfaced chemogenetics with opioid dependence. For chemogenetic experiments of ARC^AGRP^ neurons, we conducted *a priori* behavioral qualification and post-mortem FOS immunostaining verification of arcuate activation following ARC^AGRP^ chemogenetic activation. We administered clozapine during short-term and long-term morphine administration paradigms to determine the effects of dependence on food intake and body weight. We found that morphine occluded feeding behavior characteristic of chemogenetic activation of ARC^AGRP^ neurons. Notably, activation of ARC^AGRP^ neurons attenuated opioid-induced weight loss but did not evoke weight gain during opioid dependence. Consistent with these findings, we observed that morphine administration did not block fasting-induced activation of the ARC. Together, these results highlight the strength of opioidergic effects on body weight maintenance and demonstrate the utility of ARC^AGRP^ neuron manipulations as a lever to influence energy balance throughout the development of opioid dependence.

## Introduction

Upward trends in opioid abuse (Volkow et al., 2014; Scholl et al., 2018) drove the United States (U.S.) to declare a national emergency in 2017. Along with symptoms such as anxiety, low motivation, and intense withdrawal, people suffering from active opioid use disorders may also struggle with body weight-related challenges. Obesity costs the U.S. over $92 billion every year in medical expenses and imposes a huge economic burden on the population (Aljunid, 2021). Beyond the ways in which opioid use disorders can contribute to the development of obesity, obesity can also be a catalyst for opioid use when joint, nerve, and body pain caused by obesity can lead to prescription of opioids, which can lead to opioid use disorder (Stokes et al., 2019). Along with the financial burden obesity places on the United States, the opioid epidemic cost the United States over a trillion dollars in just 2017 (Luo et al., 2021).

Patients with active opioid use disorder prepare meals for themselves less often. Moreover, they prefer sweet and processed foods. However, during recovery they experience a robust appetite (Neale et al., 2012). Notably, binge eating disorders are more common in men diagnosed with opioid use disorder than those without such a diagnosis (Canan et al., 2017). Therefore, there may be clinical value in screening for eating disorders in patients with substance use disorders (Courbasson et al., 2005), as these individuals exhibit perturbed regulation of energy balance. Interestingly, previous studies in rats have shown irregular patterns of food intake and decreases in both feeding and caloric efficiency during long-term opioid exposure which caused stark weight loss (Ferenczi et al., 2010).

For humans and mice in states of energy deficit, increased activity is reported in the agouti-related peptide (AGRP) neurons (Sainsbury and Zhang, 2010) that are located in the arcuate nucleus of the hypothalamus (ARC^AGRP^) and known to orchestrate feeding behaviors (Aponte et al., 2011; Krashes et al., 2011; Mandelblat-Cerf et al., 2015; Chen et al., 2016). Moreover, these neurons indirectly trigger food intake by inhibiting brain regions involved in appetite suppression, such as the paraventricular thalamic nucleus and the parabrachial nucleus (Essner et al., 2017). Remarkably, sated mice display a maximal increase in feeding when only 800 AGRP neurons are activated (Aponte et al., 2011). Conversely, if mice are fasted, inhibition of AGRP neurons causes a reduction in food intake (Krashes et al., 2011). In addition, specific ablation of these neurons causes such a drastic reduction in body weight and food intake that starvation may result within 6 days of neuronal ablation (Wu and Palmiter, 2011). Thus, it is possible that AGRP neuronal activity could play a role in regulating feeding patterns during opioid exposure.

Opioids are known to affect brain regions implicated in reward and feeding behaviors (Romero-Picó et al., 2013). Administration of morphine on both a short- and long-term basis in mice triggered a significant up regulation of at least nine genes involved in food intake, including AGRP (Anghel et al., 2010). Moreover, previous studies showed that the administration of μ- and κ-opioid receptor antagonists attenuates food intake triggered by the activation of AGRP neurons (Brugman et al., 2002). Interestingly, a study comparing morphine effects on food intake in male and female rats and mice showed that morphine-induced weight loss was similar between the sexes (Marrazzi et al., 1996). Further research in rats has shown that heroin injection preceded a period of hypophagia, which decreased with repeated injections due to tolerance and was dependent on the dosage of heroin injected (Thornhill et al., 1976). After this period, a phase of hyperphagia was observed suggesting that heroin administration causes dysregulated feeding (Thornhill et al., 1976). In addition, rats undergoing morphine administration tended to run more quickly to a bowl of food than control rats, but ate less than controls, pointing to a multifaceted relationship between opioid administration, motivation for reward, and lack of motivation to eat (White et al., 1977). Together, these results suggest a potential complex relationship between AGRP neurons and the effects of opioids on feeding behaviors. Therefore, using chemogenetics we examined the effects of ARC^AGRP^ neuron activation on food intake and body weight during morphine dependence in mice.

## Materials and methods

### Animals

All experimental protocols were conducted in accordance with U.S. National Institutes of Health Guidelines for the Care and Use of Laboratory Animals and with the approval of the National Institute on Drug Abuse Animal Care and Use Committee. Male and female wild-type (WT; C57BL/6J background) mice were used for initial experiments characterizing the effects of morphine administration. Chemogenetic experiments were conducted with male and female heterozygous *Agrp^Cre^* mice (*Agrp^TM 1(cre)Lowl^*, C57BL/6J background; Strain # 012899, RRID:IMSR_JAX:012899; The Jackson Laboratory, ME, United States) and littermates of *Agrp^Cre^* mice lacking Cre recombinase (*Agrp^WT^*). Prior to surgery, mice were group housed with littermates in temperature and humidity-controlled rooms on a 12 h light/dark cycle with *ad libitum* access to water and rodent chow (Teklad Rodent Diet 2018, Envigo Inc., IN, United States). After surgery, mice were individually housed to accommodate food intake monitoring.

### Stereotaxic viral injections

Stereotaxic microinjection was conducted as previously described (Atasoy et al., 2008; Siemian et al., 2019). For short-term/acute morphine administration experiments, 100 nl of rAAV9/hSYN-DIO-hM3D(Gq)-mCherry, titer: 5.0 × 10^12^ GC/ml (Cat # AAV9-44361, Lot # v123022, RRID:Addgene_44361; Addgene, MA, United States) was injected bilaterally into the arcuate nucleus (ARC) of the hypothalamus of *Agrp^Cre^* mice (AP: –1.55, ML: ± 0.20, DV: –5.8 and –5.7), and *Agrp^WT^* were injected with the same Cre-dependent AAV as a control (Krashes et al., 2011). For long-term morphine administration experiments, 100 nl of rAAVrh10/hSYN-DIO-hM3D(Gq)-mCherry, titer: 5.0 × 10^12^ GC/ml (Lot # AV2517, RRID:Addgene_44361; University of North Carolina Vector Core, NC, United States) was injected unilaterally into the ARC of *Agrp^Cre^* mice (AP: –1.70, ML: + 0.20, DV: –5.8 and –5.7), and *Agrp^WT^* mice were injected with the same Cre-dependent AAV as a control for these experiments.

### Experimental timeline and procedures

#### Drugs administered

Morphine (PUBCHEM: 5288826) was obtained from the National Institute on Drug Abuse Supply Program and reconstituted with sterile saline. Ghrelin was obtained from AnaSpec (PUBCHEM: 16139313; Cat # AS-24159, AnaSpec, CA, United States) and reconstituted with sterile saline. Naloxone hydrochloride was obtained from Millipore Sigma (PUBCHEM:5464092; Cat # N7758, Millipore Sigma, MO, United States) and reconstituted with sterile saline. Clozapine was obtained from Tocris Bioscience (PUBCHEM:135398737; Cat # 0444, Tocris Bioscience, Bristol, United Kingdom) and reconstituted with sterile saline.

#### Food intake and drug administration – wild-type mice

Experiments were conducted with WT mice to describe the interaction of homeostatic feeding and manipulation of opioidergic systems. All experiments were conducted with approximately equal numbers of male and female mice.

First, a fasting-induced refeeding assay was conducted in combination with morphine administration (*n* = 8 WT mice per group). Preceding the onset of the dark cycle, mice were administered morphine subcutaneously (20 mg/kg, s.c.) and food was removed. At the onset of the light cycle, mice were administered morphine (40 mg/kg, s.c.), food was immediately provided and intake was tracked.

To test the effect of the “hunger hormone” ghrelin, we conducted a ghrelin-stimulated feeding experiment (*n* = 8 WT mice per group). Mice were administered either ghrelin (1 mg/kg, i.p.) + morphine (20 mg/kg, s.c.), ghrelin + saline, or saline + saline; food was immediately provided and intake was tracked.

For the naloxone-suppressed feeding experiment (*n* = 12–13 WT mice per group), food was removed preceding the onset of the dark cycle. At the onset of the light cycle, mice were administered naloxone (opioid receptor antagonist; 5 mg/kg, s.c.); food was immediately provided and intake was tracked.

To induce morphine dependence and characterize the effects on body weight (long-term experiment), we measured body weight changes during increasing sequential daily morphine administration (20/40/60/80/100 mg/kg, s.c.). This ascending dose schedule defines the dependence induction phase, and these doses exceed the minimal doses known to cause subsequent withdrawal (Zhu et al., 2016). Jumping, manually counted from video recordings, was quantified as a function of spontaneous withdrawal behavior on day 6 (first day of abstinence).

#### Food intake and drug administration – *Agrp^Cre^* mice

For chemogenetic experiments, a food intake qualification test was performed on all mice after recovery from AAV injection (short-term experiment: *n* = 5 WT, *n* = 10 *Agrp^Cre^*:hM3D; long-term experiment: *n* = 10 WT; *n* = 9 *Agrp^Cre^*:hM3D). This qualification consisted of a pair of daytime feeding tests under control or clozapine (CLZ) conditions. On day 1, food intake was assessed for 4 h between 10:00 A.M. and 2:00 P.M. On day 3, mice were administered CLZ (0.02 mg/kg, s.c.) at 10:00 A.M. and food intake was assessed between 10:00 A.M. and 2:00 P.M. Fold change in food intake was calculated by dividing the CLZ-induced feeding by the control day food intake. Using an *a priori* qualification threshold of 2 × food intake, 0 of 5 control WT mice qualified and 7 of 10 *Agrp^Cre^*:hM3D mice qualified for the short-term experiment; 0 of 10 control WT mice and 6 of 9 *Agrp^Cre^*:hM3D mice qualified for the long-term experiment. The three *Agrp^Cre^*:hM3D mice disqualified from each group were excluded from subsequent analyses.

For the short-term experiment (2 days after the qualification test), mice were injected with CLZ (0.02 mg/kg s.c.) and morphine (20 mg/kg s.c.) and food intake was tracked. Two days later, mice were injected with CLZ and saline and food intake was tracked.

For the long-term experiment, baseline body weight and food intake values were recorded for 3 days following the qualification test with body weight (10:00 A.M.) and food intake (4:00 P.M.) assessed daily. Body weight and food intake measurements continued daily at these time points until the experimental endpoint. Injections of CLZ (0.02 mg/kg, s.c.) were performed at these 10:00 A.M. and 4:00 P.M. timepoints. Starting on day 4, an ascending dosing schedule was implemented to define the dependence induction phase. Mice were rendered dependent on morphine via daily injection (1:00 P.M.) with an increasing daily dose over the course of 5 days (20/40/60/80/100 mg/kg, s.c.). This design was used to capture any effect of a feeding period in the morning before morphine administration, during simultaneous CLZ injection, during morphine circulation, and also during the feeding period in the evening. Starting on day 9, mice underwent a morphine dependence maintenance phase with daily injections of morphine (100 mg/kg, s.c.). Maintenance of a constant dose promotes psychomotor sensitization effects (Clark et al., 2017). Next, a morphine abstinence phase (i.e., no morphine administration) occurred for 7 days. The abstinence phase is defined by the total absence of morphine administration.

#### FOS expression analysis

*Agrp^Cre^* and control mice from the long-term experiment were injected with CLZ (0.02 mg/kg, s.c.) 90 min prior to transcardial perfusion to determine whether CLZ injection increased ARC^AGRP^ neuronal activity in the *Agrp^Cre^*:hM3D mice. For this, a quantitative analysis of FOS expression, a marker of cellular activity, was performed as described in Histology.

To determine the effects of morphine on fasting-induced activation of the ARC, WT mice were randomly split into 3 groups: fed, fasted, and fasted + morphine (*n* = 3–4 per group). Preceding the onset of the dark cycle, mice in the fasted + morphine group were administered morphine (20 mg/kg, s.c.) and food was removed. At the onset of the light cycle, fasted + morphine mice were administered morphine (40 mg/kg, s.c.) and mice in all groups were transcardially perfused for subsequent FOS histology. Fed and fasted groups were injected with saline instead of morphine and food was not removed for the fed group.

### Histology

Mice were deeply anesthetized with isoflurane and transcardially perfused with 1 × phosphate buffered saline (PBS) followed by 4% paraformaldehyde (PFA) in PBS. Whole brains were removed and post-fixed in 4% PFA at 4°C for at least 24 h before further processing. For all experiments, tissue was embedded in 4% agarose in PBS and 50-μm free-floating, coronal brain sections were collected using a vibratome (Leica VT1200S vibrating microtome, RRID:SCR_020243; Leica Biosystems Inc., IL, United States), mounted with DAPI-Fluoromount-G aqueous mounting medium (Cat # 17984-24, Electron Microscopy Sciences, PA, United States) onto Superfrost Plus glass slides (Cat # 48311-703, VWR International, PA, United States), and imaged with an AxioZoom.V16 fluorescence stereo zoom microscope (Carl Zeiss Microscopy, NY, United States).

To assess AAV expression, immunostaining was performed for mCherry using rabbit anti-DsRed polyclonal antibody (1:1,000, Cat # 632496, RRID:AB_10013483, Takara Bio USA, Inc., CA, United States). In addition, FOS immunostaining was conducted using rabbit anti-phospho-FOS monoclonal antibody (1:1,000, Cat # 5348, RRID:AB_10557109; Cell Signaling Technology, MA, United States). Slices were washed in PBS for 6 × 10 min each. Then, slices were blocked for 1 h in PBS + 0.3% Triton X-100 + 3% normal donkey serum (NDS). Samples were incubated overnight at room temperature in primary antibody diluted in block solution. The next day, slices were washed 6 × 10 min each followed by incubation in secondary antibody in block solution: goat anti-rabbit Alexa Fluor 488 (1:500, Cat # A11039, RRID:AB_2534096; Thermo Fisher Scientific, MA, United States) or donkey anti-rabbit Alexa Fluor 647 (1:500, Thermo Fisher Cat # A31573, RRID:AB_2536183). Sections were mounted onto Superfrost Plus glass slides with DAPI-Fluoromount-G mounting medium and imaged using a Keyence BZ-X710 (RRID:SCR_017202, Keyence Corp., IL, United States).

Manual cell counting was completed using the FIJI/ImageJ multi-point tool (RRID:SCR_002285; Schindelin et al., 2012). Automated cell counting was conducted using a custom ImageJ macro called Autocount. Briefly, this tool identifies each DAPI cluster as a region of interest (ROI) and measures the intensity of signal in the FOS channel within each ROI. Of note, one mouse from the long-term opioid/food intake experiment was excluded from FOS analysis because hM3D-mCherry expression was present in both hemispheres of the ARC, precluding a between-hemisphere comparison.

### Experimental design and statistical analysis

Data are reported as mean ± s.e.m. GraphPad Prism 8 software (RRID:SCR_002798; GraphPad, CA, United States) was used for all graphs and statistical analyses. Each experiment was conducted with an independent cohort of mice. For chemogenetic experiments, researchers were blinded to genotype during surgery. Student’s *t*-test was used to determine differences between groups when there were not repeated measures. For Student’s *t*-tests, Welch’s correction was applied when variance was different between two groups. Two-way repeated measures ANOVA was used to detect differences between groups and within groups when appropriate. Sphericity was not assumed and Greenhouse-Geiser corrections were made when Greenhouse-Geiser Epsilon was below 0.80. Sidak’s multiple comparisons tests were used for further evaluation when significant main effects were detected. See Supplementary Table 1 for details on all statistical analyses. The number of experimental units is depicted as “*n*” for number of animals in each experiment.

## Results

### Attenuation of food intake by administration of morphine and naloxone

**FIGURE 1:**
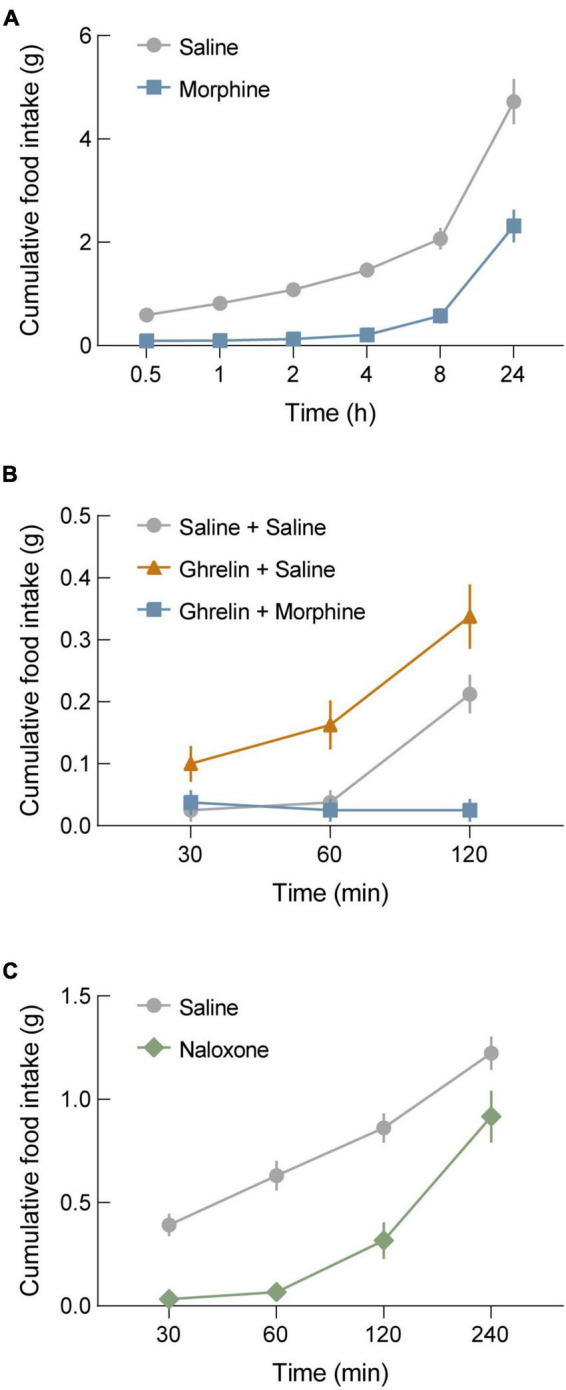
Morphine blocks both fasting-induced refeeding and ghrelin-stimulated refeeding. **(A)** Morphine administration (20 mg/kg, s.c. before fasting; 40 mg/kg, s.c. before refeeding) in wild-type (WT) mice blocks fasting-induced refeeding. **(B)** Ghrelin administration in WT mice significantly increases food intake compared to saline controls. Co-administration of morphine with ghrelin completely occludes ghrelin-stimulated feeding behavior. **(C)** Administration of the μ-opioid receptor antagonist naloxone (5 mg/kg, s.c.) blocks fasting-induced refeeding in WT mice. See Supplementary Table 1 for details on statistical analyses.

We characterized the effects of opioidergic system manipulations on food intake in wild-type (WT) mice under three different conditions. First, we used a fasting-induced refeeding paradigm plus morphine administration. For this, we fasted WT mice overnight and administered morphine at the onset of fasting (20 mg/kg, s.c.). The next morning, mice were injected again with morphine (40 mg/kg, s.c.) at the time of food reintroduction (refeeding). We found that this resulted in a significant suppression of food intake (Figure 1A), and this suppression of feeding behavior persisted for 4 h before food intake started to increase. Notably, a significant reduction of cumulative food intake remained at 24 h (Figure 1A, *p* = 0.0008). Following this, we determined the effect of morphine on ghrelin-stimulated feeding behavior. For this, WT mice were injected with either ghrelin (1 mg/kg, i.p.) + morphine (20 mg/kg, s.c.), ghrelin + saline, or saline + saline. As expected, mice administered ghrelin + saline exhibited significant increases in food intake compared to mice administered saline + saline. Conversely, mice administered ghrelin + morphine exhibited a total suppression of ghrelin-stimulated feeding behavior (Figure 1B). To further assess the effects of manipulating opioidergic systems on food intake, we again used the fasting-induced refeeding paradigm, but with injection of the opioid receptor antagonist naloxone. For this, WT mice were fasted overnight and injected with naloxone (5 mg/kg, s.c.) when food was reintroduced the following day. Interestingly, naloxone significantly suppressed fasting-induced refeeding, particularly within the first hour (Figure 1C).

### Morphine occludes ARC^AGRP^-triggered feeding behavior

**FIGURE 2:**
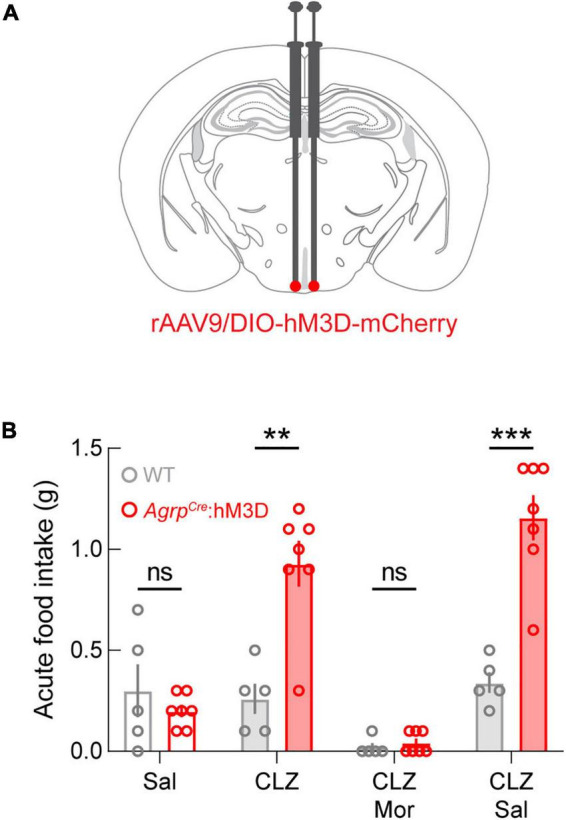
Morphine occludes ARC^AGRP^-triggered feeding behavior. **(A)** Diagram of stereotaxic microinjection into the ARC in *Agrp^Cre^* (*Agrp^Cre^*:hM3D) and *Agrp^WT^* (WT; control) mice. **(B)** While there are no differences in the feeding behavior of animals after saline administration, between groups differences are evident following clozapine (CLZ) and CLZ + saline (Sal) administration. ARC^AGRP^-triggered feeding behavior is completely abolished by morphine (Mor) administration. See Supplementary Table 1 for details on statistical analyses. **p < 0.01, ***p < 0.001.

Given the profound effect of μ-opioid receptor manipulation on homeostatic feeding, we next combined morphine administration with chemogenetic activation of AGRP neurons. For these experiments, *Agrp^Cre^* (experimental; *Agrp^Cre^*:hM3D) and *Agrp^WT^* (control; WT) mice were injected with a Cre-dependent adeno-associated virus expressing the excitatory G-protein-coupled receptor hM3D_*Gq*_ fused to the fluorophore mCherry (Krashes et al., 2011; Figure 2A). After viral transduction, we performed a qualification test to measure the well-known increase in food intake during chemogenetic activation of ARC^AGRP^ neurons (Krashes et al., 2011). For this test, sated mice were administered saline on day 1 and clozapine (CLZ; 0.02 mg/kg, s.c.) on day 3. Only experimental mice that exhibited twice as much food intake on the CLZ administration day qualified for subsequent experiments (*Agrp^Cre^*:hM3D, *n* = 7 of 10 qualified). Though control mice were subjected to the qualification test, as expected, they did not meet the qualification requirement (WT: *n* = 0 of 5 qualified). Food intake was significantly different between groups after CLZ administration but not saline administration, and food intake was significantly increased after CLZ compared to the saline administration only in *Agrp^Cre^*:hM3D mice (Figure 2B, CLZ *p* = 0.0026). Thus, these results demonstrate that the dose of CLZ administered here is sufficient to evoke food intake. On day 5, simultaneous administration of morphine (20 mg/kg, s.c.) and CLZ (0.02 mg/kg, s.c.) did not result in significant differences between groups, and on day 7, significant differences were restored between groups when mice were simultaneously administered CLZ (0.02 mg/kg s.c.) and saline (s.c.; Figure 2B, CLZ + Sal *p* = 0.0050). Notably, even the basal daytime feeding behavior observed in control WT animals was significantly lower in morphine-treated versus saline-treated mice (Figure 2B).

### Morphine dependence causes weight loss

**FIGURE 3:**
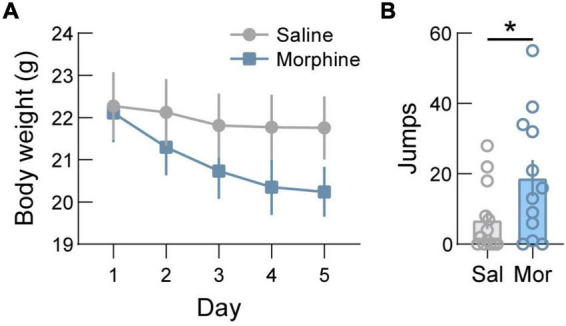
Body weight decreases after morphine dependence induction. **(A)** In wild-type mice, administration of increasing doses of morphine (20/40/60/80/100 mg/kg, s.c.) to induce dependence drives significant weight loss. **(B)** Jumping behavior, a symptomatic indicator of spontaneous withdrawal, is increased following induction of opioid dependence in saline (Sal) versus morphine (Mor) treated mice. See Supplementary Table 1 for details on statistical analyses. *p < 0.05.

Next, we validated a repeated morphine administration paradigm by measuring the effect of morphine administration on body weight and jumping as spontaneous withdrawal symptoms. For this, WT mice were injected daily with increasing doses of morphine (20, 40, 60, 80, and 100 mg/kg, s.c.) or saline. We found that this administration schedule results in a significant reduction in body weight compared to mice injected with saline (Figure 3A, *p* < 0.0001). Analysis of jumping behavior on day 6 revealed significantly more jumping in morphine administered mice compared to saline injected mice (Figure 3B, *p* = 0.0456).

### ARC^AGRP^ neuron activation attenuates opioid dependence-induced weight loss

**FIGURE 4:**
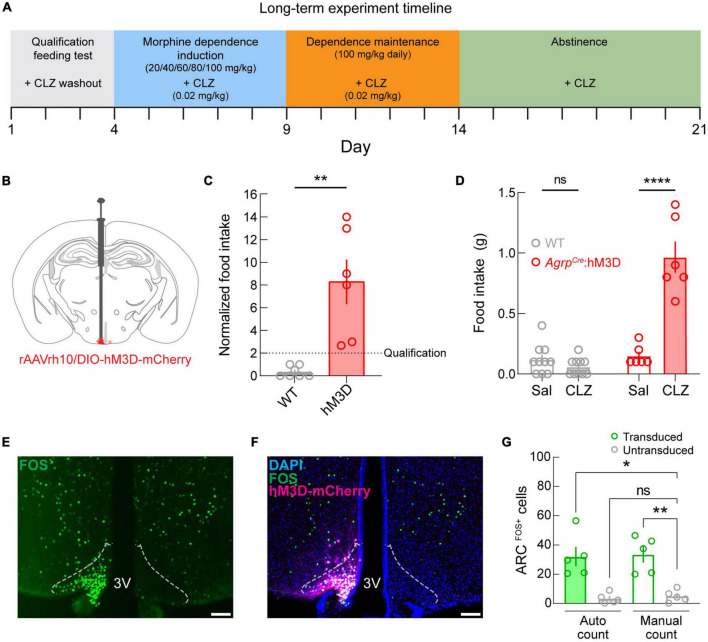
Long-term manipulation experimental design, qualification, and validation. **(A)** Diagram depicting the experimental timeline. **(B)** Diagram depicting viral microinjection into the ARC in *Agrp^Cre^* (*Agrp^Cre^*:hM3D; experimental) and *Agrp^WT^* (WT; control) mice. **(C)** Qualification of mice by normalized food intake. The dotted line denotes the qualification threshold. Experimental mice were included (*n* = 6 of 9 *Agrp^Cre^*:hM3D mice) if they consumed twice the food during the clozapine (CLZ) test compared to the saline (Sal) test. As expected, WT mice failed to meet the criterion (*n* = 0 of 10 mice) but were not excluded from further testing. **(D)** Raw food intake values for the qualification test show that food intake is increased under the clozapine condition specifically in the *Agrp^Cre^*:hM3D group. **(E)** Image of FOS immunostaining (green) showing dense fluorescently labeled cells in a single hemisphere of the ARC. Dotted outlines represent approximate ARC boundaries. Scale bar = 200 μm. **(F)** Merged image showing DAPI (blue), FOS (green), and hM3D-mCherry (magenta) expression. Dotted outlines represent approximate ARC boundaries. Scale bar = 200 μm. **(G)** Quantification of arcuate FOS expression using an automated cell counting tool (Autocount) and manual counts. 3V, third ventricle. See Supplementary Table 1 for details on statistical analyses. *p < 0.05, **p < 0.01, ***p < 0.001.

Given the clinical (Atkinson et al., 1985; Marrazzi et al., 1996; Ochalek et al., 2021) and pre-clinical (Ferenczi et al., 2010) indications at the intersection of opioid dependence and disruption of energy balance regulation, we next examined both the effects of opioid dependence on body weight and whether activation of ARC^AGRP^ neurons is sufficient to affect the changes in body weight throughout phases of opioid dependence and abstinence. For this, we used chemogenetic activation of ARC^AGRP^ neurons and measured body weight changes throughout opioid dependence induction, maintenance, and abstinence (Figure 4A).

As before, we targeted ARC^AGRP^ neurons by injecting a Cre recombinase-dependent viral vector driving the expression of hM3D_*Gq*_-mCherry (Figure 4B) into the ARC of *Agrp^WT^* (control; WT) and *Agrp^Cre^* mice (experimental; *Agrp^Cre^*:hM3D). Next, we performed the qualification test as in the previous experiment and excluded the experimental mice that did not meet the criterion (*Agrp^Cre^*:hM3D, *n* = 6 of 9 qualified). As expected, control mice did not meet the requirement (WT: *n* = 0 of 10 qualified). We observed that chemogenetic activation of ARC^AGRP^ neurons significantly increased food intake in *Agrp^Cre^*:hM3D mice compared to their control test day and control mice (Figure 4C, *p* = 0.0097), and between groups differences were detectable on CLZ days (Figure 4D, *p* < 0.0001). Moreover, multiple comparisons show that this difference is driven by the *Agrp^Cre^*:hM3D group, while no differences are detected in the control group between saline and CLZ conditions.

Post-mortem histological analysis of *Agrp^Cre^*:hM3D mouse brains was conducted to validate CLZ-mediated activation of ARC^AGRP^ neurons. We performed a quantitative analysis of FOS expression (Figure 4E) as a marker of cellular activity after injecting CLZ (0.02 mg/kg, s.c.). We observed that CLZ administration induced FOS specifically on the transduced side of the ARC in *Agrp^Cre^*:hM3D mice (Figure 4F) and found a significant increase in FOS expression in the ARC (Figure 4G, *p* = 0.0003) for the transduced hemisphere compared to the untransduced hemisphere using automated (*p* = 0.0159) and manual (*p* = 0.0069) cell counting methods.

**FIGURE 5:**
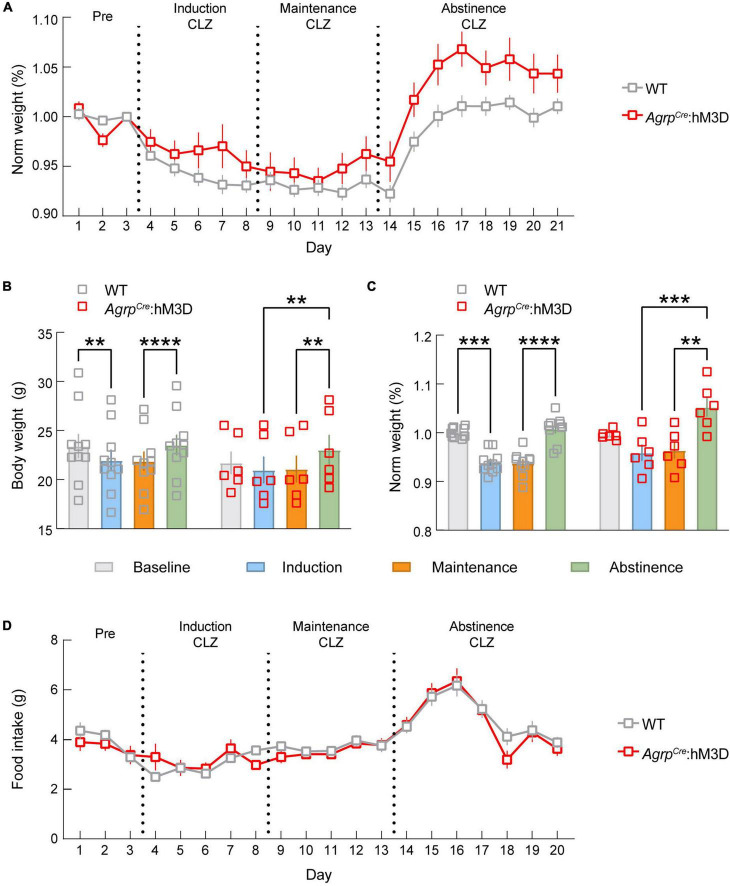
ARC^AGRP^ neuron activation attenuates morphine-induced weight loss. **(A)** Graph depicting the change in normalized body weight throughout the experiment between *Agrp^WT^* (WT; control) and *Agrp^Cre^*:hM3D groups. **(B)** Raw body weight data show that WT mice exhibit a significant drop in body weight during dependence that is not detected in the *Agrp^Cre^*:hM3D group. Both groups gain a significant amount of weight during abstinence. **(C)** Normalized body weight data show that WT mice exhibit a significant drop in body weight during dependence that is not detected in the *Agrp^Cre^*:hM3D group. Both groups gain a significant amount of weight during abstinence. **(D)** No significant differences in feeding behavior were observed throughout the duration of the experiment. See Supplementary Table 1 for details on statistical analyses. **p < 0.01, ***p < 0.001, ****p < 0.0001.

We next monitored change in energy balance and food intake during morphine dependence and abstinence. We found significant time x transgene interactions for normalized body weight (Figure 5A, *p* < 0.0001), indicating body weight change is altered by ARC^AGRP^ activation during dependence induction, maintenance, and abstinence from morphine dependence. The group differences appear to be driven by a blunting of the weight loss during the induction phase and increasing body weight during the abstinence phase. Phase effects were detectable for both groups in raw (Figure 5B, *p* < 0.0001) and normalized measures (Figure 5C, *p* < 0.0001), driven by a significant elevation of weight gain during abstinence for both groups. However, multiple comparisons of raw and normalized data demonstrate a decrease in body weight during induction (Raw *p* = 0.0013, Normalized *p* = 0.0004) and maintenance (Raw *p* = 0.0030, Normalized *p* = 0.0003) only detectable in the control group, while these differences do not exist in the experimental group. Together, these results demonstrate that ARC^AGRP^ neuronal activity is sufficient to modulate changes in body weight triggered by opioid dependence.

We also sought to determine whether activation of ARC^AGRP^ neurons modulates feeding behavior during opioid induced hypophagia. Thus, we measured food intake changes driven by chemogenetic activation of ARC^AGRP^ neurons during opioid induction, maintenance, and abstinence. Remarkably, we found that the typical increase in food intake observed during chemogenetic activation of ARC^AGRP^ neurons (Krashes et al., 2011) was not detectable during the morphine dependence or the abstinence phases (Figure 5D). This further demonstrates the morphine occlusion of ARC^AGRP^-activated feeding.

**FIGURE 6:**
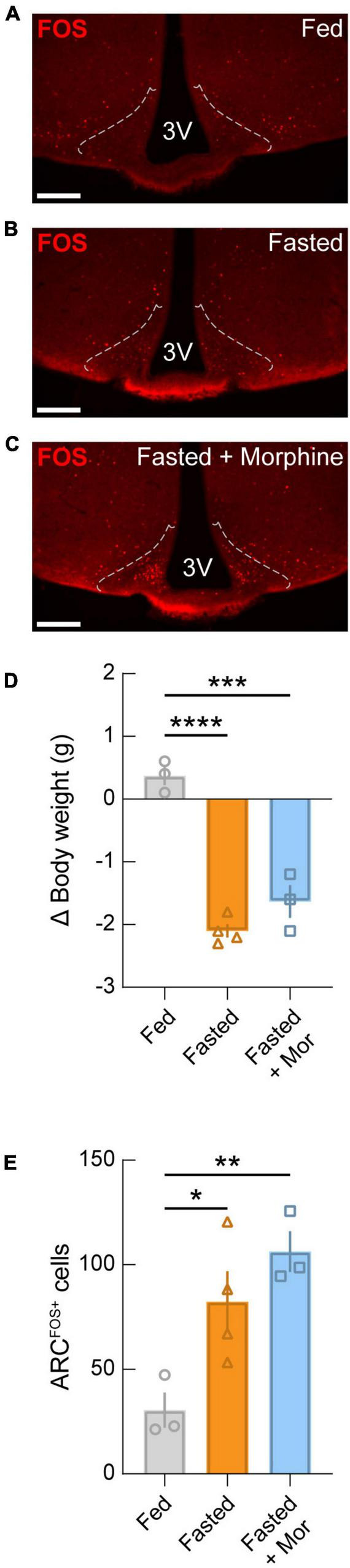
Fasting-induced activation of the arcuate nucleus is not blocked by morphine administration. Representative images of FOS immunostaining from wild-type mice that were **(A)** fed, **(B)** fasted, and **(C)** fasted with morphine administration (20 mg/kg, s.c. at the start of the fast; 40mg/kg, s.c. at the start of refeeding). Dotted lines represent approximate ARC boundaries. Scale bars = 200 μm. **(D)** Change in body weight for each condition. Significant drops in body weight occurred in both fasted and fasted + morphine (Mor) groups compared to the fed group. **(E)** Quantification of FOS-positive neurons in the ARC. Significant arcuate activation is detected in fasted mice compared to fed mice, regardless of morphine administration. See Supplementary Table 1 for details on statistical analyses. *p < 0.05, **p < 0.01, ***p < 0.001, ****p < 0.0001.

Finally, in order to determine whether morphine suppresses fasting-induced activation of the ARC, we administered morphine to mice at the onset of fasting (20 mg/kg, s.c.) and 1 h before euthanasia (40 mg/kg, s.c.) in comparison to fed and fasted mice that were not administered morphine (Figures 6A–C). We found that fasting evokes a significant loss of body weight compared to controls, regardless of morphine administration (Figure 6D). Comparable to previous reports (Laing et al., 2018), we observed that fasting induced a significant activation of the ARC which is known to be largely attributable to AGRP neuron activation. Additionally, we found that fasting-induced FOS expression was not suppressed by morphine administration (Figure 6E). Together, these results indicate that morphine does not affect fasting-induced activation of the ARC, suggesting that decreased activity in the ARC does not explain the loss of drive for feeding in opioid dependent mice.

## Discussion

Obesity and opioid dependence drive independent negative health consequences with enormous human and economic cost (Florence et al., 2016; Wang et al., 2020), and substantial overlap between obesity and opioid endemics has been demonstrated (Stokes et al., 2019). The epidemiological data showing high co-incidence of these disorders – estimated at 37% of people with obesity (Janssen et al., 2020), 1.89% of people with eating disorders (Qian et al., 2021), and 4% of people who misuse opioid prescriptions, as well as many more who obtain substances through the illicit drug market (Jannetto, 2021) – indicate a potential for a high degree of overlap. Recent work has demonstrated the critical importance of considering metabolic factors in selecting treatment for patients with opioid use disorder (Elman et al., 2020). Moreover, during opioid dependence, the selection of foods for consumption becomes limited. Our findings reveal a potential therapeutic role for ARC^AGRP^ neurons in mediating the overlap between food intake and body weight maintenance during morphine dependence.

Our work may also have clinical relevance because of the ability of ARC^AGRP^ neuronal activation to attenuate opioid-induced weight loss, thereby offsetting the suppression of food intake by prescription opioids. Importantly, our results also highlight the post-use abstinence phase as a period of caloric surplus where humans suffering from opioid use disorder may experience repeated cycles of weight loss and weight gain that could lead to deleterious effects on body composition marked by replacement of skeletal muscle with fat mass. Consistent with this theory, humans suffering from opioid use disorder show greater rates of obesity and unhealthy eating habits (Ochalek et al., 2021).

Previous studies have also reported interactions between both regulatory homeostatic and opioidergic systems. For example, resistance to high fat diet-induced obesity is observed in mice lacking δ-opioid receptors (Czyzyk et al., 2012) and μ-opioid receptors (Tabarin et al., 2005). Furthermore, blockade of κ-opioid receptors promotes weight loss (Cintron-Colon et al., 2019). Together, these findings suggest that homeostatic regulation of metabolism is intertwined at multiple levels with opioidergic systems. Thus, the implications of this interface may drive phenomena such as naloxone blockade of a move-eat-move behavioral food-seeking paradigm that reduces the latency for neuropeptide Y (NPY) to drive feeding behavior without changing total food intake (Rudski et al., 1996). Previous studies have shown that NPY action in the paraventricular nucleus of the thalamus (PVN) drives feeding behavior via the nucleus solitary tract in a naltrexone-dependent manner (Kotz et al., 2000). Furthermore, antagonism of opioid receptors by naloxone opposes feeding and AGRP-mediated increase in preference for high fat diet in rats (Hagan et al., 2001); we replicated this finding in mice. In addition, we showed that the characteristic increase in food intake triggered by the activation of ARC^AGRP^ neurons (Krashes et al., 2011) is occluded by acute and long-term morphine use. The mechanisms for these seemingly paradoxical actions of morphine and naloxone on feeding are unclear to us. Perhaps downstream neuronal circuits regulated by the activity of ARC^AGRP^ neurons are affected by naloxone and morphine similarly or by independent mechanisms. Given that we observe fasting-induced activation of arcuate neurons regardless of morphine administration, the suppression of feeding behavior by morphine could occur at downstream effectors of AGRP neurons. It is likely that the ARC^AGRP^ neurons are unable to override the morphine- and naloxone-driven states, perhaps due to locomotive effects in the morphine state and affective states driven by naloxone.

Compulsive drug use relates to sensitization of ventral striatal dopamine circuitry (Evans et al., 2006). AGRP-impaired mice exhibit enhanced dopamine in the forebrain and spike-timing dependent long-term potentiation with an altered set point for reward circuitry (Dietrich et al., 2012). Opioids increase dopamine release from the ventral tegmental area to the ventral striatum, which would be associated with a temporary decrease in reward value for food (Low et al., 2021). During abstinence-induced withdrawal and precipitated withdrawal in rodents, ventral striatal dopamine levels are markedly reduced (Acquas et al., 1991; Pothos et al., 1991). Thus, the ventral striatal dopamine increase that occurs upon cessation of AGRP neuron activity during food intake could result in large phasic reward signals that slow the onset of satiety (Reichenbach et al., 2021). This phenomenon is reflected in our data showing fluctuations on body weight and food intake throughout phases of morphine dependence. While the effect of chemogenetic activation of ARC^AGRP^ neurons is insufficient to completely override the attenuation of food intake and weight gain during morphine dependence, there is a detectable difference that could be beneficial. Thus, it may be important to consider that approaches for stimulation of ARC^AGRP^ activation, such as scheduled food intake (Laing et al., 2018) and exercise (Bunner et al., 2020), may have limited efficacy toward increasing motivation for feeding during dependence. The blunted effect of chemogenetic activation of ARC^AGRP^ neurons may be partially attributable to changes to the inhibitory control of AGRP neurons that have been reported to occur during sustained Gq signaling independent of weight gain (Ewbank et al., 2020). However, it is noteworthy that even in acute experiments and on the first day of the long-term morphine administration experiment there was not a significant increase of feeding behavior. Thus, it is likely that competition from a variety of mechanisms influenced the effects reported here. For now, regardless of the mechanism, the implication is that there is limited utility of sustained ARC^AGRP^ neuron activation using chemogenetics for opioid users. However, co-administration of strategies to activate ARC^AGRP^ neurons, such as exercise (Landry et al., 2022), may limit the harm by opioids on patient weight maintenance for individuals prescribed opioids.

At the simplest level, it could appear surprising that food intake evoked by ARC^AGRP^ neuron activation is occluded by morphine administration. If they were independent mechanisms, the potent effect of ARC^AGRP^ neurons to drive feeding should oppose the potent effect of morphine to drive weight loss. However, our results suggest that feeding evoked by the activation of ARC^AGRP^ neurons relies on functions in competition with morphine’s targets or downstream effectors. Alternatively, it is possible that changes in peripheral inputs, such as withdrawal-related stomach discomfort (De Schepper et al., 2004; Leppert, 2015), may override ARC^AGRP^ neuron activation. Interestingly, the effects do not seem to be mediated by the “hunger hormone” ghrelin, given that ghrelin-stimulated feeding behavior is completely occluded by morphine. Nevertheless, our findings highlight the potency of opioids to control food intake and override the effects of ARC^AGRP^ neurons on feeding. While we did not measure energy expenditure in this study, and previous reports demonstrated that ARC^AGRP^ neuron activation dramatically reduces expenditure (Krashes et al., 2011) and substrate preference (Ewbank et al., 2020), the major effect of morphine administration in our study was on suppression of food intake.

One limitation to this study is that the sample size is relatively small for the long-term experiment. This is particularly true in the experimental group and was a function of subject exclusion that was incorporated into the experimental design. Notably, the number of experimental mice included in this study is consistent with previous reports of chemogenetic activation of ARC^AGRP^ neurons (Krashes et al., 2011; Dietrich et al., 2012). Group size differences partially account for the lack of significant detection of body weight loss in the experimental group as they still lost weight. Remarkably, during dependence, mice did not gain weight when ARC^AGRP^ neurons were activated during morphine administration. However, there may also be an effect on body weight gain. During abstinence, there is a large effect in the opposite direction whereby the *Agrp^Cre^*:hM3D mice outgained the control group during activation of ARC^AGRP^ neurons. Hence, further studies are needed to interface neuronal populations that regulate metabolism with drugs of abuse. Furthermore, while our descriptive experiments show that the suppression of feeding behavior by morphine is not a sex-dependent effect, not enough male and female mice passed qualification to conduct a sufficiently powered analysis of sex differences (Huhn et al., 2018). Given that naltrexone has beneficial effects for weight management in female but not male mice (Atkinson et al., 1985), formal investigation into sex hormone interactions with ARC^AGRP^ neuron activation and opioid dependence are justified. However, these three-way interactions were outside the scope of the current study. In addition, it is unclear if differences between rodent and human ventral striatal function during withdrawal (Shokri-Kojori et al., 2021) may influence changes in body weight and food intake following dependence. Given that there is no additive effect of ARC^AGRP^ neuron activation on food intake during the abstinence phase, it is possible that the activation of these neurons is a functional component of post-opioid dependence weight gain. Our results showing the lack of suppression of arcuate activity by morphine administration support the premise that the rebound from opioid suppressed feeding may include excessive AGRP neuron activation. Formal inquiry about the activity patterns of ARC^AGRP^ neurons throughout the stages of dependence will yield insight into the ideal phase to inhibit these neurons during abstinence induced weight gain. Finally, it is important to consider this experiment from a harm-reduction perspective to focus on improving physiological regulation during dependence rather than changing the drug consumption.

In summary, our work cautions limited utility of ARC^AGRP^ neuron activation as a potential lever to influence body weight via changes in food intake during opioid use. Our results show volatile periods of body weight fluctuations during opioid dependence induction and abstinence that may be critical for management of body weight maintenance during opioid use disorders. Treatment of opioid dependence should coincide with interventions that encourage behavioral management of energy balance, including ARC^AGRP^ neuron activation or its downstream effectors. Together, our findings will serve as a basis for future experiments investigating how drugs of abuse affect hypothalamic circuitry to influence energy balance.

## Data availability statement

The raw data supporting the conclusions of this article will be made available by the authors, without undue reservation.

## Ethics statement

This animal study was reviewed and approved by the National Institute on Drug Abuse Animal Care and Use Committee.

## Author contributions

BL and YA: conceptualization and writing – original draft. BL, AJ, and DW: data curation. BL, AJ, LE, and AP: formal analysis. All authors: writing – review and editing.
