# Supplementary material for: Regulation of body weight and food intake by AGRP neurons during opioid dependence and abstinence in mice
Source: Front Neural Circuits. 2022 Aug 30;16:977642. doi: 10.3389/fncir.2022.977642 (PMC9468932; doi:10.3389/fncir.2022.977642)
Supplement: Supplementary file 1 [file Table_1.DOCX]

**Supplementary Table 1. Statistical analysis details and values**

| **Figure** | **Test Name** | **Assumptions** | **Test Statistics** | **Test p Value** | **Multiple Comparisons** | **p Value** |
| --- | --- | --- | --- | --- | --- | --- |
| Fig 1A | Two-way RM ANOVA,  n = 8 per group | QQ plot approximately normal, residuals approximately normal, homoscedasticity plot approximately normal | Time x treatment: F(6, 138) = 18.17  Time: F(1.158, 26.64) = 168.7  Treatment: F(1, 23)= 44.65  Mouse: F(23,138) = 5.202 | p < 0.0001  p < 0.0001  p < 0.0001  p < 0.0001 | Between groups  *Fast vs. Fast + Morphine*  0.5hr  1hr  2hr  4hr  8hr  24hr | p < 0.001  p < 0.001  p < 0.001  p < 0.001  p < 0.001  p = 0.0008 |
| Fig 1B | Two-way RM ANOVA,  n = 8 per group | QQ plot approximately normal, residuals approximately normal, homoscedasticity plot approximately normal | Time x treatment: F(4, 42) = 22.02  Time: F(1.438, 30.19) = 74.64  Treatment: F(2, 21) = 11.97  Mouse: F(21, 42) = 8.438 | p < 0.0001  p < 0.0001  p = 0.0003  p < 0.0001 | Between groups  *0-30min*  G/S vs. S/S  G/S vs. G/M  S/S vs. G/M  *30-60min*  G/S vs. S/S  G/S vs. G/M  S/S vs. G/M  *60-120min*  G/S vs. S/S  G/S vs. G/M  S/S vs. G/M | p = 0.0918  p = 0.1716  p = 0.8681  p = 0.0325  p = 0.0191  p = 0.8681  p = 0.0932  p = 0.0005  p = 0.1213 |
| Fig 1C | Two-way RM ANOVA,  n = 13 saline,  n = 12 naloxone | QQ plot approximately normal, residuals approximately normal, homoscedasticity plot approximately normal | Time x treatment: F(3, 69) = 3.654  Time: F(1.588, 36.52) = 122.6  Treatment: F(1, 23) = 31.70  Mouse: F(23, 92) = 5.363 | p < 0.0166  p < 0.0001  p < 0.0001  p < 0.0001 | Between groups  *Fast vs. Fast + Naloxone*  0.5hr  1hr  2hr  4hr | p < 0.0001  p < 0.0001  p = 0.0002  p = 0.1604 |
| Fig 2B | Two-way RM ANOVA,  n = 6 WT mice,  n = 6 hM3D | QQ plot approximately normal, residuals approximately normal, homoscedasticity plot approximately normal | Treatment x Transgene: F(3, 30) = 15.36  Treatment: F(2.180, 21.80) = 30.99  Transgene: F(1, 10) = 33.99  Mouse: F(10, 30) = 1.068 | p < 0.0001  p < 0.0001  p = 0.0002  p = 0.4145 | Between groups  Sal  CLZ  MOR CLZ  CLZ Sal  Within Groups  *Cre+*  Sal vs. CLZ  Sal vs. MOR + CLZ  Sal vs. CLZ + Sal  CLZ vs. MOR CLZ  CLZ vs CLZ Sal  MOR + CLZ vs CLZ + SAL    *Cre-*  Sal vs. CLZ  Sal vs. MOR + CLZ  Sal vs. CLZ + Sal  CLZ vs. MOR CLZ  CLZ vs CLZ Sal  MOR + CLZ vs CLZ + SAL | p = 0.9339  p = 0.0026  p = 0.9022  p = 0.0050  p = 0.0028  p = 0.0315  p = 0.0004  p = 0.0019  p = 0.7818  p = 0.0002  p > 0.9999  p = 0.5188  p > 0.9999  p = 0.1807  p = 0.9835  p = 0.0061 |
| Fig 3A | Two-way RM ANOVA,  n = 16 saline,  n = 14 morphine | QQ plot approximately normal, residuals approximately normal, homoscedasticity plot approximately normal, GG corrected due to GG epsilon = 0.4613 | Day x Group: F(4, 112) = 25.04  Day: F(1.845, 51.66) = 85.14  Group: F(1, 28) = 0.9542  Mouse: F(28, 112) = 443.0 | p < 0.0001  p < 0.0001  p = 0.3370  p < 0.0001 |  |  |
| Fig 3B | Student’s t-test,  n = 12 saline,  n = 13 morphine (experimental error; one run not video recorded) | Independent samples approximately normally distributed | t _23_ = 2.114 | p = 0.0456 | - | - |

| Fig 4C | Welch’s t-test, n = 10 WT,  n = 6 hM3D | Heterogeneity test failed, p < 0.0001 | t_5.084_ = 4.029 | p = 0.0097 | - | - |
| --- | --- | --- | --- | --- | --- | --- |
| Fig 4D | Two-way RM ANOVA,  n = 10 WT,  n = 6 hM3D | QQ plot approximately normal, residuals approximately normal, homoscedasticity plot approximately normal | Treatment x Transgene: F(1,14) = 44.23  Treatment: F(1,14) = 32.95  Transgene: F(1,14) = 91.33  Mouse: F(1,14) 0.5528 | p <0.0001  p < 0.0001  p < 0.0001  p = 0.8603 | Between groups (WT-hM3D)  Ctrl  Clz  Within groups (Ctrl-CLZ)  WT  hM3D | p = 0.9203  p < 0.0001  p = 0.7187  p < 0.0001 |
| Fig 4G | Two-way RM,  n = 5 matched pairs | QQ plot approximately normal, residuals approximately normal, homoscedasticity plot approximately normal | Treatment: F(1.945, 7.779) = 28.65  Mouse: F(4, 12) = 5.299 | p = 0.0003  p = 0.0108 | U-A vs. T-A  U-A vs. T-M  U-A vs. U-M  U-M vs. T-M  U = untransduced  T = transduced  A = Autocount  M = manual count | p = 0.0159  p = 0.0088  p = 0.1504  p = 0.0069 |
| Fig 5A | Two-way RM ANOVA,  n = 10 WT,  n = 6 hM3D | QQ plot approximately normal, residuals approximately normal, homoscedasticity plot approximately normal | Time x Transgene: F(4.010, 57.35) = 63.56  Time: F(20, 286) = 3.680  Transgene: F(1,17) = 5.216 | p < 0.0001  p < 0.0001  p = 0.6093 | Within groups between days  *WT*  PreD3 vs. Day 1 and 2  PreD3 vs. Day4 to Day 14  PreD3 vs. Day 15 to 21    *hM3D*  PreD3 vs. Day 11  PreD3 vs. All other days  Between groups  All days | p > 0.9999  p < 0.0003  p > 0.5000  p = 0.0615  p > 0.05  p > 0.05 |

| Fig 5B | Two-way RM ANOVA,  n = 10 WT,  n = 6 hM3D | QQ plot approximately normal, residuals approximately normal, homoscedasticity plot approximately normal | Phase by transgene: F(3, 42) = 3.956  Phase: F(1.879, 26.31)= 47.04  Transgene: F(1,14): 0.3319  Mouse: F(14,42) = 179.3 | p = 0.0143  p < 0.0001  p = 0.5737  p < 0.0001 | Within groups between phases  *WT*  Baseline vs. Induction  Baseline vs. Sensitization  Baseline vs. Abstinence  Induction vs. Sensitization  Induction vs. Abstinence  Sensitization vs Abstinence  *hM3D*  Baseline vs. Induction  Baseline vs. Sensitization  Baseline vs. Abstinence  Induction vs. Sensitization  Induction vs. Abstinence  Sensitization vs. Abstinence  Between groups within phases  Baseline  Induction  Sensitization  Abstinence | p = 0.0013  p = 0.0030  p = 0.9649  p = 0.9542  p < 0.0001  p < 0.0001  p = 0.2366  p = 0.3112  p = 0.1327  p = 0.3303  p = 0.0018  p = 0.0030  p = 0.0008  p = 0.0017  p = 0.5746  p = 0.2500 |
| --- | --- | --- | --- | --- | --- | --- |
| Fig 5C | Two-way RM ANOVA,  n = 10 WT,  n = 6 hM3D | QQ plot approximately normal, residuals approximately normal, homoscedasticity plot approximately normal | Phase by transgene: F(3, 42) = 4.152  Phase: F(1.879, 26.31)= 61.97  Transgene: F(1,14): 0.0902  Mouse: F(14,42) = 5.634 | p = 0.0115  p < 0.0001  p = 0.0902  p < 0.0001 | Within groups between phases  *WT*  Baseline vs. Induction  Baseline vs. Sensitization  Baseline vs. Abstinence  Induction vs. Sensitization  Induction vs. Abstinence  Sensitization vs. Abstinence  *hM3D*  Baseline vs. Induction  Baseline vs. Sensitization  Baseline vs. Abstinence  Induction vs. Sensitization  Induction vs. Abstinence  Sensitization vs. Abstinence  Between groups within phases  Baseline  Induction  Sensitization  Abstinence | p = 0.0004  p = 0.0003  p = 0.8279  p = 0.9898  p < 0.0001  p < 0.0001  p = 0.1980  p = 0.2685  p = 0.1008  p = 0.2855  p = 0.008  p = 0.018  p = 0.8561  p = 0.7625  p = 0.5746  p = 0.2500 |

| Fig 5D | Two-way RM ANOVA,  n = 10 WT,  n = 6 hM3D | QQ plot approximately normal, residuals approximately normal, homoscedasticity plot approximately normal | Day x transgene: F(19,266) = 1.342  Day: F(4.857, 68.00) = 33.90  Transgene: F(1, 14) = 0.08910  Mouse: F(14,266) = 14.53 | p = 0.1569  p < 0.0001  p = 0.7697  p < 0.0001 | Within groups between days  *WT*  Day 3 vs. Day 4 to 13  Day 3 vs. Day 14 to 17  Day 3 vs. Day 18 to 20    *hM3D*  Day 3 vs. Day 4 to 14 & 16  Day 3 vs. Day 15 & 17  Day 3 vs. Day 18 to 20  Between groups within days  All days | p > 0.05  p < 0.0221  p > 0.05  p > 0.05  p < 0.0207  p > 0.05  p > 0.05 |
| --- | --- | --- | --- | --- | --- | --- |
| Fig 6D | One-way ANOVA,  n = 3 fed,  n = 4 fasted,  n = 4 fasted + morphine | QQ plot approximately normal, residuals approximately normal, homoscedasticity plot approximately normal | Treatment: F(2,7) = 58.02 | p < 0.0001 | Fed vs. Fasted  Fed vs. Fasted + Morphine  Fasted vs. Fasted + Morphine | p < 0.0001  p = 0.0003  p = 0.1899 |
| Fig 6E | One-way ANOVA,  n = 3 fed,  n = 4 fasted,  n = 4 fasted + morphine | QQ plot approximately normal, residuals approximately normal, homoscedasticity plot approximately normal | Treatment: F(2, 7) = 8.909 | p = 0.0119 | Fed vs. Fasted  Fed vs. Fasted + Morphine  Fasted vs. Fasted + Morphine | p = 0.0198  p = 0.0045  p = 0.2248 |
